# Effectiveness of telemedicine systems for adults with heart failure: a meta-analysis of randomized controlled trials

**DOI:** 10.1007/s10741-019-09801-5

**Published:** 2019-05-24

**Authors:** Ye Zhu, Xiang Gu, Chao Xu

**Affiliations:** 1 Clinical Medical College of Yangzhou University, Yangzhou, 225001 Jiangsu China; 2 Department of Cardiology, Northern Jiangsu People’s Hospital, Yangzhou, China; 3 Department of Biostatistics and Epidemiology, University of Oklahoma Health Science Center, Oklahoma City, OK 73104 USA

**Keywords:** Meta-analysis, Heart failure, Telemedicine, Cardiovascular disease, Randomized controlled trials

## Abstract

Despite favorable effects from telemedicine (TM) on cardiovascular diseases, outcome and comparative impact of TM on heart failure (HF) adults remain controversial. A meta-analysis was conducted to summarize the evidence from existing randomized controlled trials (RCTs) which compared potential impact of TM on HF with conventional healthcare. TM mainly included structure telephone support (STS), involving interactive vocal response monitoring and telemonitoring. PubMed, MEDLINE, EMBASE, and the Cochrane Library were searched to identify RCTs to fit our analysis (1999 to 2018). Odds ratio (OR) with its 95% confidence interval (CI) was used. Sensitivity analysis, subgroup analysis, and tests for publication bias were conducted. Heterogeneities were also evaluated by *I*^2^ tests. A total of 29 RCTs consisting of 10,981 HF adults were selected for meta-level synthesis, with follow-up range of 1–36 months. Telemonitoring is associated with the reduction in total number of all-cause hospitalization (OR 0.82, 95% CI 0.73–0.91, *P* = 0.0004) and cardiac hospitalization (OR 0.83, 95% CI 0.72–0.95, *P* = 0.007). Telemonitoring resulted in statistically significant risk reduction of all-cause mortality (OR 0.75, 95% CI 0.62–0.90, *P* = 0.003). However, the OR of HF-related mortality (OR 0.84, 95% CI 0.61–1.16, *P* = 0.28) is not significantly distinguishable from that of conventional healthcare. Receiving STS interventions is likely to reduce the hospitalization for all causes (OR 0.86, 95% CI 0.78–0.96, *P* = 0.006, *I*^2^ = 6%) and the hospitalization due to HF (OR 0.74, 95% CI 0.65–0.85, *P* < 0.0001, *I*^2^ = 0%), compared with interventions from conventional healthcare. OR of all-cause STS mortality (OR 0.96, 95% CI 0.83–1.11, *P* = 0.55) was identified in meta-analyses of eight cases. OR of STS cardiac mortality (OR 0.54, 95% CI 0.34–0.86, *P* = 0.009) was identified in meta-analyses of three cases. This work represents the comprehensive application of network meta-analysis to examine the comparative effectiveness of telemedicine interventions in improving HF patient outcomes. Compared with conventional healthcare, telemedicine systems with medical support prove to be more effective for HF adults, particularly in reducing all-cause hospitalization, cardiac hospitalization, all-cause mortality, cardiac mortality, and length of stay. While further research is required to confirm these observational findings and identify optimal telemedicine strategies and the duration of follow-up for which it confers benefits.

## Introduction

Heart failure (HF) is one of the most prevalent manifestations of cardiovascular disorders [1], decreasing the health-related quality of life (QoL) of HF patients while increasing the burden of morbidity, mortality, and healthcare. As the result of the rising demand for acute hospital beds for HF, strategies to facilitate early discharge and reduce unplanned readmissions are superior to improving patient outcomes and resource usage. Poor adherence to recommendations provided by healthcare professional is responsible for ~ 50% of HF hospitalization [2]. The overall purpose of education and other intervention modalities, for instance, structured telephone support (STS), home visits, and nurse-led clinics, is to improve self-care and patients’ adherence. In recent years, promising results have been reported for multidisciplinary care strategies for HF patients with and without telemedicine systems. A meta-analysis has shown positive results for home-based interventions, including a reduction of 34% in all-cause mortality and a reduction of 30–56% in HF hospitalizations [3]. However, following prospective randomized multicenter clinical trials of non-invasive approaches cannot confirm these findings for morbidity-related and mortality-related endpoints [4, 5]. Although earlier studies suggest a reduction in mortality, results of the study reported by Chaudhry and colleagues failed to show any beneficial effects of telemonitoring [6].

To determine whether telemedicine systems improve outcomes for adults following an unplanned admission due to HF, we conducted a meta-analysis of existing randomized controlled trials (RCTs) comparing different forms of telemedicine systems with conventional healthcare after discharge. Interventions included conventional healthcare and the following forms of telemedicine: STS involving regular follow-up calls between the health professional and the patient, telemonitoring systems involving the transmission of information on symptoms and signs (TM), and telemedicine systems involving interactive vocal response monitoring and electrocardiographic transmissions (ECG). The primary endpoints of this study were all-cause hospitalization and all-cause mortality during follow-up. As secondary endpoints, we explored cardiac hospitalization, mortality, and length of stay.

## Materials and methods

### Search strategy

A search of literature was performed for RCTs of non-invasive telemedicine systems for HF patients compared with conventional healthcare. The PubMed, MEDLINE, EMBASE, and the Cochrane Library databases were searched to extract articles (1999 to 2018) on telemedicine systems in adults. Literature search was informed using keywords heart failure, cardiac failure, remote, telemedicine, telecommunication, telehealth, telecardiology, health information systems, internet, home monitor, and interactive voice response. The meta-analysis of RCTs was performed in accordance with the latest meta-analysis guidelines (PRISMA) [7]. Referenced studies and narrative reviews were searched as well.

### Study selection

Studies involved in this meta-analysis were sorted by three independent authors. In the preliminary stage, all publication titles and abstracts were examined by the first two authors to exclude non-pertinent articles clearly not meeting the following inclusion criteria. The two authors reviewed the results of each study with a standardized data extraction tool and also applied standard scales to judge study quality and risk of bias independently. If any doubt of suitability remained after the abstract was examined, the full manuscript was retrieved and assessed. Most of the disagreements were resolved through discussions. When there was any disagreement, the third author mediated the discussion to gain consensus.

### Inclusion criteria

- Studies were included in the meta-analysis if they were RCTs.
- Patients were objectively confirmed to have symptomatic HF (New York Heart Association [NYHA] Class I–IV) characterized by impaired left ventricular function (left ventricular ejection fraction < 45%).
- Includes both an experimental group and a control group.
- Telemedicine treatments include telephone support, telemonitoring involving interactive vocal response monitoring, and monitoring by ECG.
- Control group only receives conventional healthcare defined as a guideline-based standard care with scheduled clinic visits but without any additional interventions.
- The primary outcome measurements include all-cause mortality and all-cause hospitalization (defined as an admission to a healthcare facility for less than 24 h for all causes).
- The secondary outcome measurements include cardiac hospitalization (defined as an admission to a healthcare facility for less than 24 h due to heart failure), cardiac mortality, length of hospital stay, health-related QoL, and hospitalization costs.

### Exclusion criteria

Studies were excluded if they met the following criteria:

- Not being RCTs and non-English language papers
- Not involving patients with acute HF
- Not reporting numerical data on the outcomes of interest
- Published in the form of letters, congress abstracts, review articles, comments, editorials, case reports, technical reports, or animal studies

### Data extraction

A predesigned data abstraction form was used to obtain data on relevant results of the study. Following terms were recorded for each study: authors, years of publication, patient demographic data (gender, age, disease severity, etc.), intervention features, outcomes parameters, as well as the quality of included RCT studies. Detailed information would be requested from the author if some necessary original data could not be acquired. Data were then tabulated and a network meta-analysis (NMA) of the following outcomes was deemed appropriate: all-cause mortality, all-cause hospitalization, cardiac hospitalization, and cardiac mortality. Studies satisfying the inclusion criteria were assigned a quality score based on the revised 7-point Jadad scale [8]. The scale consists of four aspects: generation of allocation sequence (2 points), allocation concealment (2 points), investigator blindness (2 points), and withdrawals and dropouts (1 point). A total score of less than 4 indicates low quality, while the one of more than 5 indicates high quality [9].

### Statistical analysis

Separate analyses were performed for each outcome’s odds ratio (OR) or weighted mean difference (WMD) using the Mantel–Haenszel method. Pairwise meta-analyses were conducted by combining studies comparing the same interventions using a random-effects model. Meta-analysis inconsistency was assessed by comparing the deviance and deviance information criteria in fitted consistency and inconsistency models across studies [10]. Specifically, we investigated the heterogeneity through examining both forest plots and Cochran’s *Q* quantified by *I*^2^ tests [11]. An *I*^2^ of 0–25% indicates no heterogeneity, an *I*^2^ of 25–50% indicates moderate heterogeneity, an *I*^2^ of 50–75% indicates large heterogeneity, and an *I*^2^ of 75–100% indicates extreme heterogeneity. Results with a *P* value less than 0.05 and 95% confidence intervals (CIs) exceeding 1 were considered as statistical significance. The analyses were carried out using Comprehensive Meta-Analysis techniques in Review Manager (RevMan, version 5.2, The Cochrane Collaboration, London, England, 2012) [12]. The results from our network meta-analysis were qualitatively compared with direct, frequent, pairwise estimates. Publication bias was tested by funnel plots and the Egger and Begger tests using Stata version 12.0 software (Stata Corporation, College Station, TX, USA) and *P* < 0.05 was considered significant [13].

## Results

### Trial flow

**Fig. 1:**
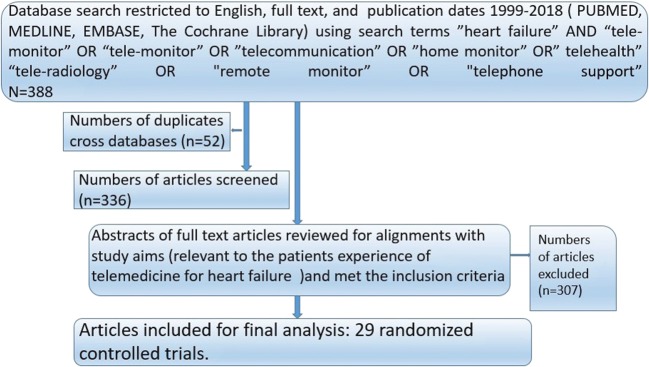
Selection process of the studies

As shown in Fig. 1, 388 citations were identified from our search (up to August 2018). Fifty-two duplication cross-databases were excluded. Three hundred eight were excluded after examining titles and abstracts of full-text articles. Reasons for exclusion were not related to HF, not RCT, unrelated to home-based telemonitoring/telephone support, no outcome of interest, or non-English language papers and so forth. From the remaining articles, we identified 29 non-duplicated RCTs and 10,981 patients eligible for the meta-analysis. Details about the searching strategy and the flow chart for the identification of studies used in the network meta-analysis of telemedicine interventions for HF patients were provided in Fig. 1.

### Characteristics of included trials

General characteristics of the population, interventions, and comparison groups included in the 29 RCTs along with the main outcomes of each study were summarized in Table 1. All the RCT studies were classified into two groups based on the type of telemedicine intervention(s): telemonitoring (*n* = 19) and telephone-supported systems (*n* = 9). Please note that only one study reported outcomes for both telemonitoring and telephone-supported care. The average duration of the interventions was 10.5 months (range 1 to 36 months). For most of the studies (25 out of 29), the number of males was greater than that of females. Endpoints and adopted telemedicine strategies were similar among the selected studies. In 22 of 29 trials, participants were followed for six or more months. Despite differences in the scope and range of included studies, most RCTs reported on a number of similar outcomes. Most frequently reported outcomes included all-cause hospitalization, cardiac hospitalization, all-cause mortality, and cardiac mortality. Other commonly reported outcomes comprised the impact of telemedicine interventions on quality of life, length of hospital stay, as well as hospitalization costs. Acceptability, patient satisfaction, and emergency room visits were rarely reported in the studies and therefore were excluded from our final analysis. In most of the trials, interventions were typically delivered by nurses. Using the revised 7-point Jadad scale, all the selected RCTs had Jadad scores greater than 3, which suggested a good study design and high study quality. A more detailed description of included trials is provided in Table 1.

**Table 1:** Description of included studies

Author/year	Study population			Population	Type of interventions	Follow-up lengths	Outcome parameters	Jadad score
	*N*	Age (year)	Female (%)					
Ewa Hägglund/2006	72	75 ± 8	5	Sweden	Home intervention versus usual care.	4 months	Health-related quality of life (HRQoL), hospital days due to HF	5
Silvia Soreca/2012	118	≥ 70	49	Italy	Clinical and electrocardiographic evaluations and periodic home echocardiographic examinations versus usual care	18 months	1. Rehospitalization for worsening of heart failure symptoms and/or for the appearance of major vascular events 2. Home-treated vascular events, cardiovascular death, and the composite endpoint of death plus rehospitalization	5
Abul Kashem/2006	36	56.1 ± 12.6	30.5	America	Telemedicine arm versus usual care	8 months	1. Total hospital days 2. Effect of outpatient monitoring on duration of carvedilol titration	4
Abul Kashem/2008	48	53.6 ± 2.6	25	America	Telemedicine group versus usual care	1 year	Office visits, emergency department visits, hospitalization, telephone calls	4
S Scalvini/2005	230	59 ± 9		Italy	Home-based telecardiology versus usual care	1 year	Readmission due to heart failure; cardiovascular events	4
Jeffrey A. Spaeder/2006	49	54.5	33	America	Telemedicine system versus usual care	3 months	Adverse events	5
William T Abraham/2011	560	61	27	America	A wireless implantable hemodynamic monitoring system versus usual care	6 months	Heart failure-related hospitalizations	5
Sarwat I. Chaudhry/2010	1653	61	42	America	Telemonitoring of interactive voice response system versus usual care	6 months	1. Readmission for any reason hospitalization for heart failure, number of days in the hospital, and number of hospitalizations	6
Friedrich Koehler/2011	710	66.9 ± 10.7	19	Germany	Remote telemedical management versus usual care	26 months	1. Death from any cause 2. A composite of cardiovascular death and hospitalization for HF	5
Christine S. Ritchie/2016	346	63.2 ± 13	48.5	America	A care transition nurse (CTN), interactive voice response versus usual care	1 month	1. 30-day rehospitalization 2. (1) Rehospitalization and death, (2) days in the hospital and out of the community	5
Josiane J.J. Boyne/2012	382	71 ± 11	41	Netherland	Telemonitoring versus usual care	1 year	1. Mean time to first heart failure-related hospitalization 2. Heart failure admission and all-cause mortality	5
A. Giordano/2009	455	57 ± 10	15	Italy	Home-based telemanagement versus usual care	24 months	1. All-cause hospital readmissions 2. Mean cost for hospital readmission	5
P. Dendale/2012	160	76 ± 10	35	Belgium	Telemonitoring versus usual care	6 months	1. All-cause mortality 2. Hospitalization costs	5
Andrew Weintraub/2010	188	69	34	America	Telemonitoring versus usual care	3 months	1. HF hospitalization 2. Heart failure inpatient days 3. Quality of life	5
Goldberg/2003	280	59 ± 15	32	America	Alere net system versus usual care	6 months	1. Hospitalization rates 2. Mortality	4
Marcia J. Wade/2011	316	78.1	47.7	America	Telemonitoring versus usual care	6 months	Emergency department visits, hospital admissions, and death	5
Patrik Lynga/2012	344	73 ± 10.2	25	Sweden	Telemonitoring versus usual care	12 months	Hospitalization and death	6
Roberto Antonicelli/2008	57	70	38.5	Italy	Telemonitoring versus usual care	16 months	Mortality and rate of hospitalization, quality of life, and costs	5
Gallagher BD/2017	40	64 (50–77)	25	America	Telemonitoring versus usual care	1 month	Readmission and adherence	5
Claudio Pedone/2015	90	80 ± 7	61.2	Italy	Telemonitoring and telephone versus usual care.	6 months	All-cause death and hospital admissions	5
Henry Krum /2013	405	73 ± 10	37	Australia	Usual care and telephone support versus usual care	12 months	The Packer clinical composite score; hospitalization for any cause	4
GESICA /2005	1518	65	29	Argentina	Telephone versus usual care	16 months	Mortality and quality of life	5
Lynda Blue/2001	165	75 ± 8	69	Britain	Telephone versus usual care	1 year	HF hospitalization and mortality	5
Ann S. Laramee/2003	287	70.7 ± 11.8	28	America	Telephone versus usual care	90 days	Readmission rate and readmission cost	6
Fernanda B. Domingues/2010	111	63 ± 13	32	Brazil	Telephone versus usual care	3 months	Rehospitalizations and deaths	5
Daniel Ferrante/2010	1518	65 ± 13.3	29.2	Argentina	Telephone versus usual care	3 years	Rate of death or hospitalization	5
Robert Frank DeBusk/2004	462	72 ± 11	52	America	Telephone versus usual care	1 year	Rate of rehospitalization	5
Wendy A/1999	181	67.2	32.0	America	Telephone versus usual care	6 months	All-cause mortality; heart failure mortality	5
Edward K./2002	200	63.5	39.5	America	Telephone versus usual care	6 months	Hospital readmissions and mortality; quality-of-life score	5

### Health-related outcomes and meta-analysis

**Fig. 2:**
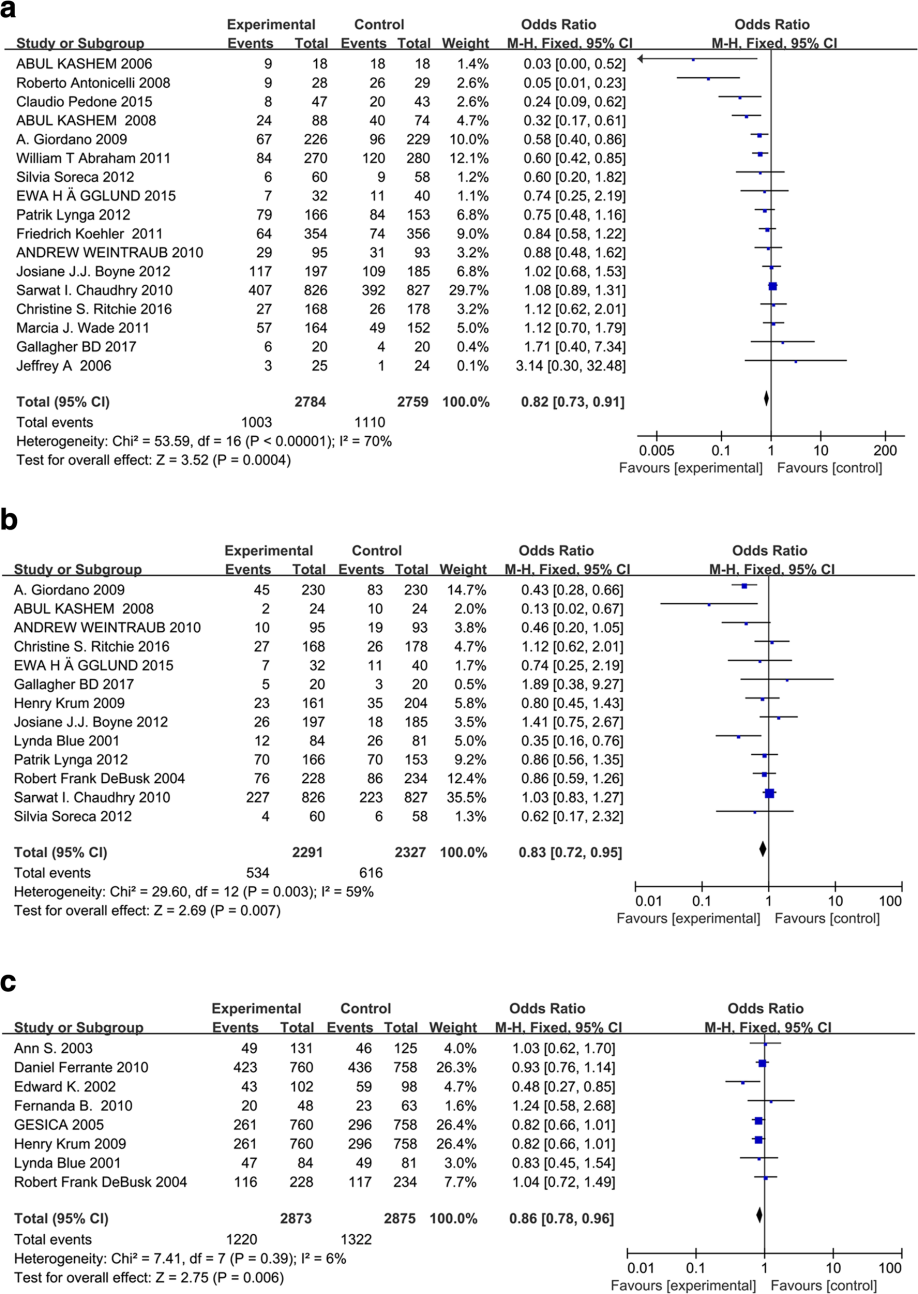

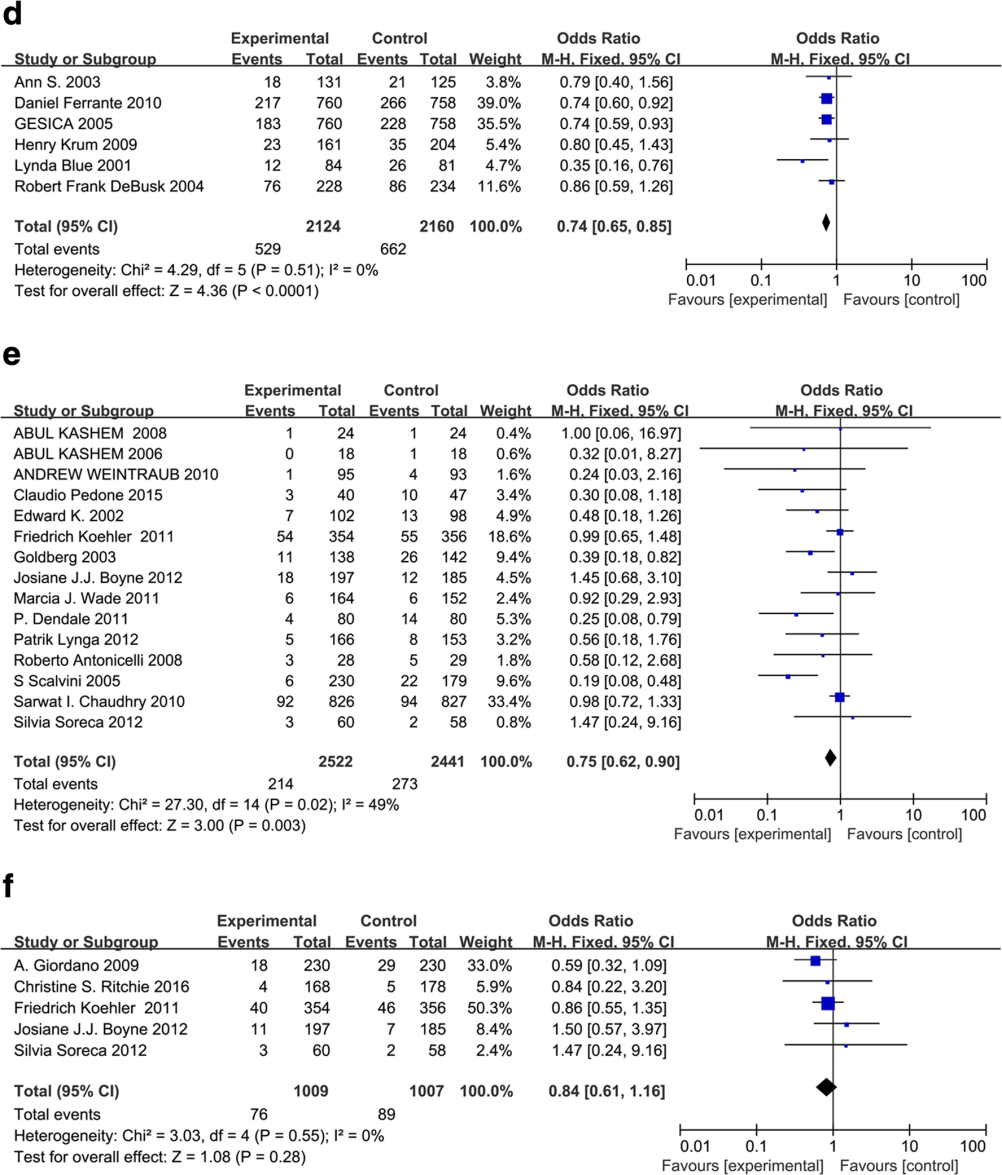

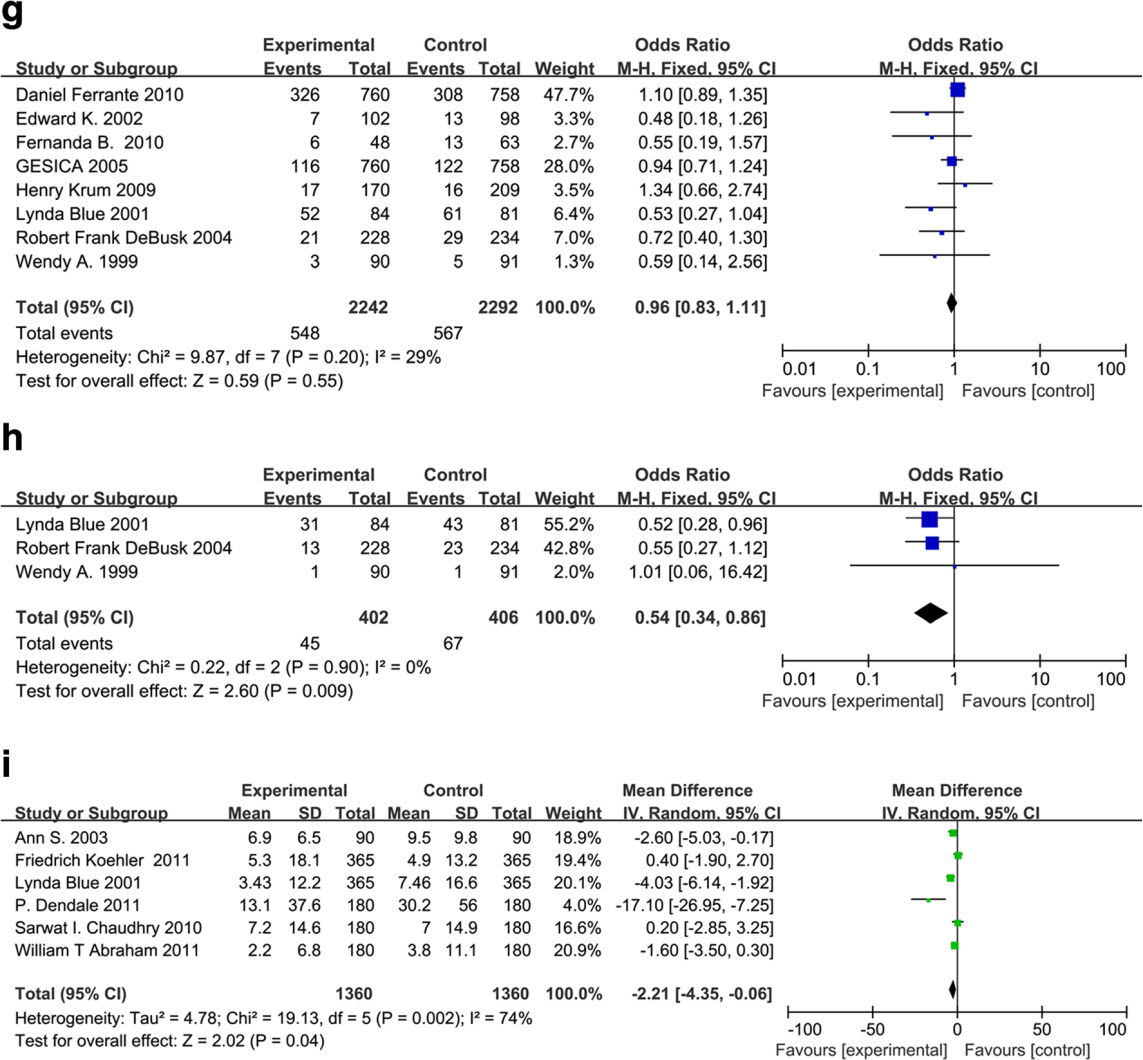
**a** Effect of telemonitoring versus usual care on all-cause hospital admission in patients with chronic heart failure. CI, confidence interval; M-H, Mantel–Haenszel. **b** Effect of telemonitoring versus usual care on cardiac hospital admission in patients with chronic heart failure. CI, confidence interval; M-H, Mantel–Haenszel. **c** Effect of telephone support interventions versus usual care on all-cause hospital admission in patients with chronic heart failure. CI, confidence interval; M-H, Mantel–Haenszel. .**d** Effect of telephone support interventions versus usual care on cardiac hospitalization in patients with chronic heart failure. CI, confidence interval; M-H, Mantel–Haenszel. **e** Effect of telemonitoring versus usual care on all-cause mortality in patients with chronic heart failure. CI, confidence interval; M-H, Mantel–Haenszel. **f** Effect of telemonitoring versus usual care on cardiac mortality in patients with chronic heart failure. CI, confidence interval; M-H, Mantel–Haenszel. **g** Effect of telephone versus usual care on all cause of mortality in patients with chronic heart failure. CI, confidence interval; M-H, Mantel–Haenszel. **h** Effect of telephone versus usual care on cardiac mortality in patients with chronic heart failure. CI, confidence interval; M-H, Mantel–Haenszel. **i** Effect of interventions versus usual care on length of hospital stay in patients with chronic heart failure. M-H = Mantel–Haenszel risk ratio. Data are from full peer-reviewed publications only and reflect the most recent meta-analysis of telemedicine in heart failure

**Fig. 3:**
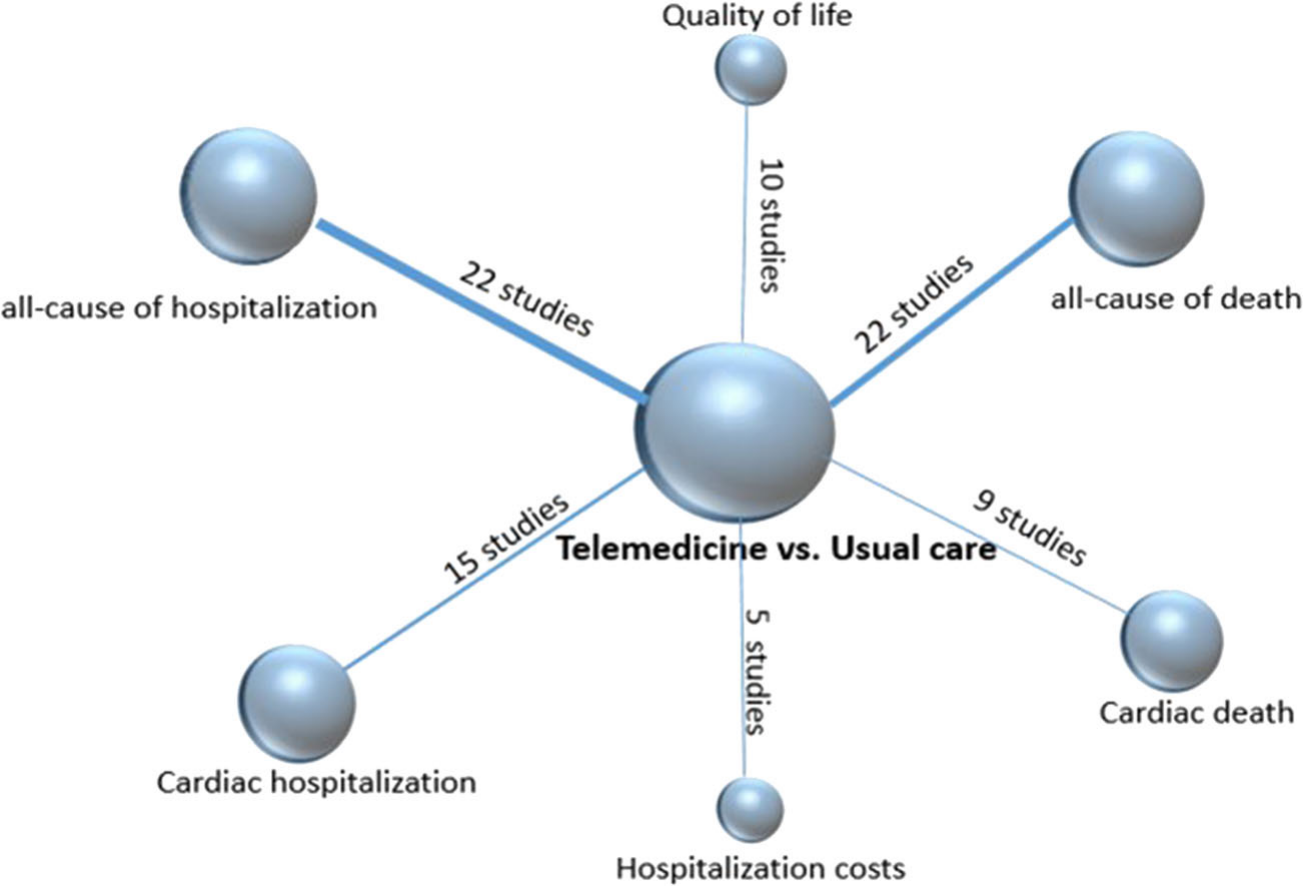
Evidence network for interventions included in the analysis of the outcomes of telemedicine versus usual care. Each node represents different outcomes and the size of each node indicates the total number of studies included in the network

All-cause hospitalization was reported in 23 studies and cardiovascular diseases-related hospitalization was reported in 16 studies. With respect to clinical outcomes, 22 trials contributed to the analysis of the all-cause death and 9 trials analyzed the death due to heart failure. We did the meta-analyses for the outcomes of all-cause of hospitalization, all-cause of mortality, cardiac hospitalization, and cardiac mortality (Fig. 2). Evidence network for interventions included in the analysis of the outcomes of telemedicine versus conventional healthcare was shown in Fig. 3.

### All-cause hospitalization and cardiac hospitalization

With respect to all-cause hospitalizations, most studies reported beneficial effects of different telemedicine system interventions. Telemonitoring was associated with a reduction in a total number of all-cause hospitalization (OR 0.82, 95% CI 0.73–0.91, *P* = 0.0004). The effect was statistically significant for 17 out of 19 studies. Heterogeneity was moderate (*I*^2^ = 70%; Fig. 2a), which is supposed to result from individual discrepancies (age, gender distribution, etc.) and interventions (methods, duration, etc.). Two studies reported HF readmission within 30 days [14, 15]. Thirty-day rehospitalization rates were similar in the telemonitoring intervention group and the UC control group. Cardiac hospitalizations were reported in 13 studies. The OR of telemonitoring versus conventional healthcare on cardiac hospitalization was 0.83 (95% CI 0.72–0.95, *P* = 0.007), suggesting a significant difference between the two groups (Fig. 2b). Compared with conventional healthcare, structured telephone support interventions reduced the hospitalization for all causes (OR 0.86, 95% CI 0.78–0.96, *P* = 0.006, *I*^2^ = 6%) and due to HF (OR 0.74, 95% CI 0.65–0.85, *P* < 0.0001, *I*^2^ = 0%) (Fig. 2c, d).

### All-cause of mortality and cardiac mortality

A significant reduction in the risk of all-cause mortality (OR 0.75, 95% CI 0.62–0.90, *P* = 0.003) was identified in meta-analyses of 15 studies of telemonitoring. However, the OR of telemonitoring versus conventional healthcare on cardiac mortality was not significant (OR 0.84, 95% CI 0.61–1.16, *P* = 0.28) in a meta-analysis of five studies. The heterogeneity of these two analyses was moderate (*I*^2^ = 49%; Fig. 2e) and none (*I*^2^ = 0%; Fig. 2f), respectively. No significant effect of telephone support intervention on all-cause mortality (OR 0.96, 95% CI 0.83–1.11, *P* = 0.55) was identified in meta-analyses of eight studies (Fig. 2g). A significant effect of telephone support intervention on cardiac mortality (OR 0.54, 95% CI 0.34–0.86, *P* = 0.009) was identified in meta-analyses of studies (Fig. 2h).

### Length of hospital stay

HF-related length of stay was reported in 13 studies comparing interventions with conventional healthcare [16–28], including 3 telephone-supported studies and 10 telemonitoring studies. There was a significant heterogeneity when data from the telemedicine interventions studies were pooled (*df* = 5, *P* = 0.002, *I*^2^ = 74%, as shown in Fig. 2i). The analysis revealed that there was a significant difference in HF-related length of hospital stay between the overall interventions and control groups (pooled standardized difference in means = − 2.21, 95% CI − 4.35 to − 0.06, *Z* = 2.02, *P* = 0.04), the telemonitoring and control groups (pooled standardized difference in means = − 1.71, 95% CI − 4.83 to − 1.42, *Z* = 1.07, *P* = 0.28), or the telephone-supported care and control groups (pooled standardized difference in means = − 3.41, 95% CI − 5.01 to − 1.82, *Z* = 4.2, *P* = 0.0001). William et al. [16] reported that the length of hospital stay for HF-related hospitalizations was significantly shorter in the treatment group than in the control group (2.2 days [SD 6.8] vs. 3.8 days [SD 11.1], *P* = 0.02) during 6 months follow-up. However, no significant difference in HF-related length of hospital stay was observed comparing the telemonitoring group with the control group (pooled standardized difference in means = − 1.71, 95% CI − 4.83 to − 1.42, *Z* = − 1.07, *P* = 0.28).

### Publication bias

**Fig. 4:**
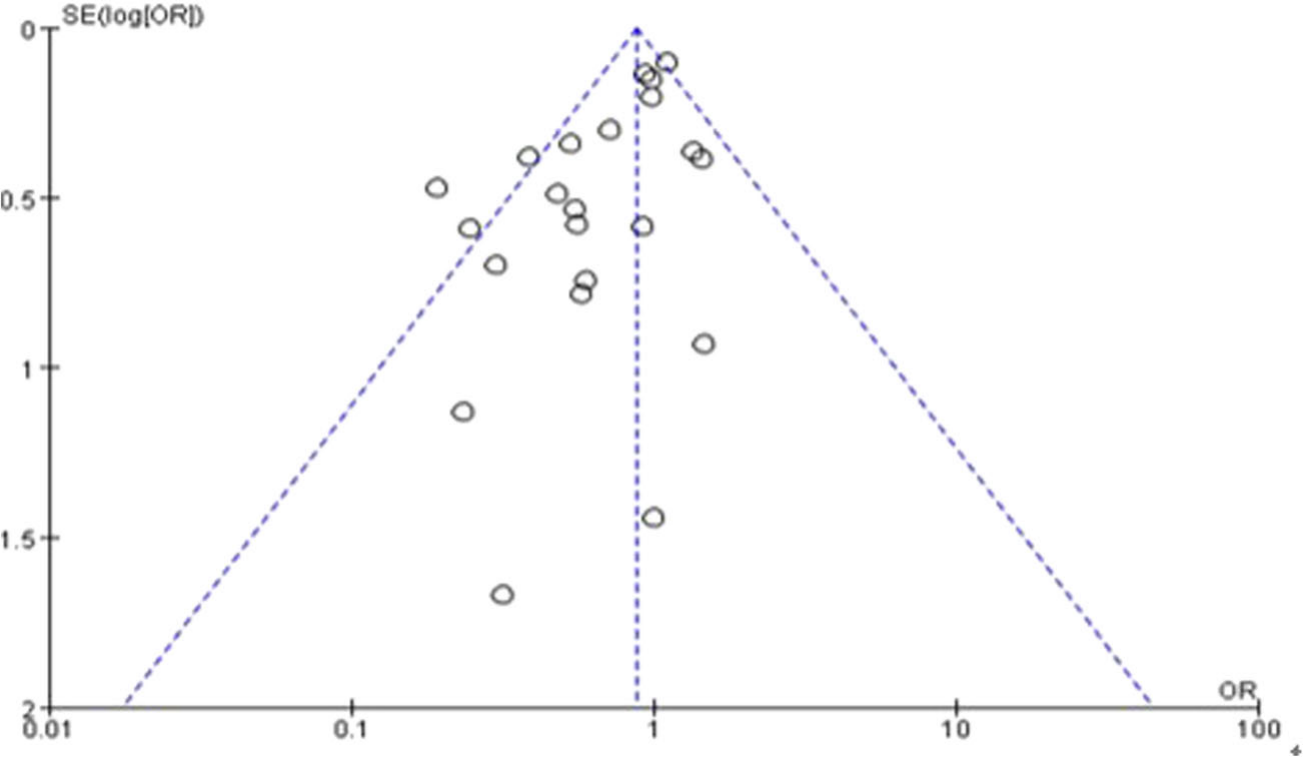
Funnel plot comparing interventions versus controls reporting all-cause mortality. Funnel plot assessing publication bias

Funnel plots and Egger’s testing were performed to assess the publication bias of all of the studies. There was no significant evidence of publication bias for all-cause mortality of telemedicine interventions, which was revealed by the Egger and Begger tests (Egger: *P* = 0.888; Begger: *P* = 0.582). The funnel plot did not display asymmetry, while both Egger’s and Begg’s test indicate no publication bias (Fig. 4).

### Quality of life

Ten telemedicine studies reported slight improvements in measures of QoL for HF adults receiving telemedicine as compared to those receiving conventional healthcare [18, 22, 25, 26, 28–33]. Quality of life was measured by various questionnaires, including the Minnesota Living with Heart Failure Questionnaire (MLHFQ), score for SF-36 and SF-12, and the Kansas City Cardiomyopathy Questionnaire (KCCQ), making it difficult to compare the outcomes. However, patients in the telemedicine group were more likely to report stable or deteriorated symptoms, compared with those in the control group. SF-36 was applied in two studies. One study found [18] that patients randomly allocated to the remote telemedical management (RTM) group showed an improved score for SF-36 physical functioning over the entire study period (*P* < 0.05) compared with the conventional healthcare group. Ewa et al. [22] observed the improvements in health-related QoL using disease-specific measures of the KCCQ: the telemedicine group had significantly higher score than control group over 3 months follow-up (*P* < 0.05). Three studies contributed to the analysis of the MLHFQ, while the SF-12 was also applied in three studies. Goldberg reported that among patients completing their 6-month follow-up visit, patients in both groups experienced improvement between baseline and 6 months in their Minnesota Living with Heart Failure, SF-12, and Health Distress scores. Although no difference was statistically significant, the intervention group tended to improve all the quality of life measures [30]. Additionally, GESICA [31] found that the intervention group had a significantly better quality of life than the conventional healthcare group (mean total score on MLHFQ (30.6 vs. 35, *P* = 0.001).

### Hospitalization costs

Five studies [16, 19, 26, 27, 34] examined the effects of telemedicine interventions on hospitalization costs. Although hospitalization costs were reported in the five original studies, different applied methodologies did not allow the pooled results into the meta-analysis. The general outcomes were statistically inconclusive and varied depending on the context and specific telemedicine systems of the studies. Furthermore, many studies showed that there was no significant difference in hospitalization costs between intervention groups and non-intervention groups. Dendale et al. [19] reported that even though the total hospitalization cost for HF was almost doubled in the control group (1458 + 3420 Euro/patient) compared with the telemedicine group (902 + 2277 Euro/patient), the difference was not significant (*P* = 0.23). Laramee et al. reported that the intervention did not increase costs and no significant differences were found in both outpatient and inpatient resource utilization between the groups [27].

## Discussion

In this meta-analysis, we performed an up-to-date assessment of the effectiveness of telemedicine systems for the management of HF patients. By summarizing the current best evidence, this network meta-analysis showed that compared to conventional healthcare, telemedicine intervention appears to be beneficial for patients with HF, particularly in reducing all-cause hospitalization, cardiac hospitalization, all-cause mortality, cardiac mortality, and length of stay in HF patients. This work represents the comprehensive application of network meta-analysis to examine the comparative effectiveness of telemedicine interventions in improving HF patient outcomes.

Available studies examining the patient experience and effect of patient education on acceptance and adherence to the intervention of telemonitoring were limited [34, 35]. The adoption of telemedicine is driven by the expectation that it should improve patient outcomes and reduce healthcare costs. Few studies, however, have compared telemedicine with conventional health care with respect to healthcare utilization [36]. In a recent network meta-analysis of RCTs by Kotb et al. [37], teletransmission was found to reduce the odds of mortality as well as the HF-related hospitalizations compared with the conventional healthcare. In the present meta-analysis of 29 studies, we have specifically considered planned or unplanned hospital visits that provide additional data to the field with an updated literature search up to 2018. Most of the evidence that is currently available on the impact of telemedicine interventions involves the comparison of an active form of telemedicine to conventional healthcare. Findings from this network meta-analysis are unique in that various comparisons were examined across different forms of telemedicine interventions. Most available literature focused on the primary outcomes of mortality and hospitalizations. We have found that telemedicine is associated with a significant reduction in the total number of hospital visits and mortality. In other words, telemedicine safely reduces healthcare utilization by reducing elective face-to-face hospital visits.

HF is a complex illness, and optimal management requires regular patient monitoring. However, the financial and organizational strain on healthcare systems prevents timely monitoring frequently. This could lead to reliance on patient help-seeking which often occurs when it is too late to prevent hospitalization [38]. Essentially, telemedicine is a diagnostic modality which, without an appropriate therapeutic intervention, could not be expected to alter clinical outcomes. If one assumes that the appropriateness of such interventions is similar with or without telemedicine, the only possible advantage of telemedicine is shortening the time to a treatable, or “actionable,” event. In this respect, we should consider that none of the telemedicine systems should be expected to predict some events, e.g., acute cardiac death or no cardiac death. As was reported by Sarwat et al. [17], non-implantable telemonitoring systems for HF do not seem to improve outcomes compared with conventional healthcare. Being the largest RCT done so far, a comprehensive non-invasive telemonitoring system did not reduce morbidity or mortality in 1653 patients who were randomly assigned to the telemonitoring system versus conventional healthcare. It is still unclear which factor could explain the significant reduction in mortality achieved by telemedicine [6]. The inconsistent outcomes of the telemedicine program may be due to the lack of consensus protocol or guideline for conducting telemedicine care. The purpose of a remote interview may range from improving diet and treatment compliance to regular monitoring of the HF-related symptoms and self-management. However, many home monitoring systems are designed for transmission of body weight, blood pressure, and heart rate via a standard telephone line or network system to a central server. It may be helpful to monitor the real-time clinical condition of the patients for early treatment.

The previous network meta-analysis included 21 studies which included a control group and examined the impact of telemonitoring. Finally, only nine of them were followed participants for more than 6 months [19]. In this meta-analysis, most of the studies had longer than 6 months of follow-up. This may suggest that the potential benefits of telemedicine require longer periods of follow-ups before they are observed. Telemedicine is part of a comprehensive package of care that includes education and empowerment of the patients, early warning of deterioration, access to health professionals’ advice and moral support, and pharmacological intervention. For instance, one of the studies reported [39] that remote monitoring with an automated telemedicine system can successfully facilitate titration of carvedilol in outpatients with class II and III HF defined by NYHA. Telemedicine titrations were as successful as titrations in the clinic. Further, the time to reach the final dose of carvedilol was significantly shorter in the intervention group compared to that of conventional healthcare group (33.6 vs. 63.7 days, *P* < 0.001). Telemedicine interventions, therefore, reflect complex healthcare strategies and are not limited to simple data-gathering.

Positive results were found on health-related QoL in patients with chronic HF, although data were limited. Indeed, QoL is an important measure of health, particularly for older people and those suffering from HF. Maintaining moderate or a high and improving level of physical activities is associated with a better health-related QoL in patients with chronic HF [40]. Regarding the quality of the studies included in this meta-analysis, different questionnaires were used (SF-36, SF-12, MLHF, KCCQ), making it difficult to compare outcomes. However, the majority demonstrated improvements in the patients who underwent telemedicine intervention, especially in terms of the MLHFQ and SF-36 physical aspects, and these improvements led to both better life quality and favorable prognoses. Further information is required to assess the effect of the telemonitoring use on the patient’s QoL, and perceptions of health status, as this was cited as a barrier against uptake and adherence. This network analysis was limited to RCTs only. This was deemed appropriate, however, given the availability of a substantial amount of evidence and the reduced likelihood of bias and confounding associated with this study design. For the most part, the risk of bias associated with included studies was found to be either low or moderate and further sensitivity analyses did not significantly differ from the main analysis of the study.

With constrained resources for healthcare expenditures, it is reasonable to expect an evaluation of new technologies not just on safety and efficacy but also on cost-effectiveness. With the substantial societal and economic impacts of HF, telemedicine interventions that can help minimize the likelihood of cost of care associated with HF may provide significant benefits to the healthcare system. Researchers that used an analytical approach for assessing healthcare and patient-related costs showed that most of the telemedicine was cost saving as the telemedicine groups have shorter “stay of length.” There is no consensus on hospitalization costs for HF patients. Some differences across models may be expected. However, many reports showed that there was no significant difference in hospitalization cost use of the intervention and conventional healthcare groups [27].

Admittedly, our meta-analysis has its limitations. Telemedicine interventions were heterogeneous in terms of monitored parameters and HF selection criteria. Our study depended only on the data reported in studies, some endpoint data were unavailable, and considering the limited number of studies, publication and reporting biases were inevitable to some extent. It is difficult to testify differences between intervention duration and intervention designs. These differences in study designs resulted in low to large scores of heterogeneity (0% to 75%). Furthermore, some trials were underpowered to detect the primary outcome and did not report outcome assessor blinding. Telemedicine usually builds on self-monitoring, with evidence that it can help educate patients about which symptoms and signs are most important and what measures can be taken to destabilize the syndrome. The content of telemedicine interventions was often poorly described, making it difficult to understand exactly what was provided. Moreover, due to the differences in the selection criteria of the included studies (e.g., LVEF and New York Heart Association), the generalizability of the treatment effect is unclear.

## Conclusions

Telemedicine interventions appear to lead to benefits for patients with CHF, decrease all-cause hospital admissions, and improve QoL, although there are still several important issues to consider. Only limited studies are available on the cost-benefits and appropriate business models for the interventions; impacts of these interventions on patient QoL have only been reported in a few studies, and the optimal duration of these interventions is still not clear. There is also a significant lack of researchers concentrating on HF patients in remote rural areas who might benefit from a telemedicine service. Further research is therefore required to fill in this gap in knowledge. We suggest that other aspects should also be addressed, as telemedicine is not the only one component of managing HF and could not replace face-to-face consultations between healthcare providers and patients. Such studies need to investigate, from the patients’ perspective, the effect of educational methods and technological supports, benefits of tailored monitoring, and cost-effectiveness analysis. The efficacy of telemedicine using an advanced telemonitoring device and newly developed guidelines in the remote follow-up and management of HF patients should also be investigated.
